# The Development of a Novel Device Based on Loss of Guidewire Resistance to Identify Epidural Space in a Porcine Model

**DOI:** 10.1155/2020/8899628

**Published:** 2020-08-22

**Authors:** Jaeyoung Yang, Rakmin Choi, Kyubum Cho, Seonjin Kim, Bousung Lee, Kwanghaeng Lee, Dongseok Kim, Jaedo Lee

**Affiliations:** ^1^Department of Anesthesiology and Pain Medicine, Veterans Health Service Medical Center, Seoul, Republic of Korea; ^2^Department of Anesthesiology and Pain Medicine, Armed Forces Capital Hospital, Seongnam, Republic of Korea

## Abstract

**Background:**

The application of additive manufacturing (3D printing) has been recently expanded to various medical fields. The new technique named loss of guide wire resistance (LOGR) was developed via 3D printing for the detection of epidural space using a guide wire instead of air or saline used in the loss of resistance (LOR) technique.

**Methods:**

The prototype model of epidural space finder consists of a polyactic acid (PLA) or a resin. It was manufactured with 3D printing. Biocompatibility test (eluate and sterility tests) was performed in both products. The advantage of the newly developed device was compared with conventional loss of resistance (LOR) technique in a porcine model.

**Results:**

Eluate and sterility tests revealed that the PLA was more biocompatible than the resin. The LOGR technique facilitated rapid access to epidural space compared with the LOR technique (41.64 ± 32.18 vs. 92.28 ± 61.46 seconds, *N* = 14, *p*=0.0102, paired sample *t*-test), without any differences in success rate (87.5%).

**Conclusion:**

We conclude that LOGR technique is comparable to LOR technique to access the epidural space, although the advantage of either technique in terms of complications such as dural puncture or epidural hematoma is unknown. We demonstrated the potential benefit of 3D printer for the development of a new medical device for anesthesia.

## 1. Background

Additive manufacturing, also known as three-dimensional printing, is a rapid prototyping technology. Three-dimensional solid objects can be generated from a digital model designed by a computer. It provides a huge opportunity for rapid manufacture of medical implants, surgical planning, and education [1]. In medical field, the most significant advance in 3D printing application mainly relates to surgery, especially in surgical planning of facial surgery, neurosurgery, and cardiothoracic and orthopedic surgeries [2–5]. Three-dimensional printing was used for the manufacture of prostheses in orthopedics [6]. The 3D printed anatomical model based on radiographic images was used to educate surgeons and students [7, 8]. In anesthesiology, 3D printing is used for ultrasound training or planning of tracheal intubation [9, 10], We explored the role of 3D printing for the development of medical devices in anesthesiology. We report the use of 3D printing for the development of epidural space finder, made of polyactic acid (PLA) or resin. The principle is based on the release of guidewire resistance of the new epidural space finder when the needle is inserted into the epidural space. A biocompatibility test was conducted to determine the biological safety of 3D printing products. The usefulness of the newly developed device was compared with conventional loss of resistance technique in a porcine model. The PLA model was more biocompatible than the resin model. The newly developed epidural space finder (loss of guidewire resistance technique (LOGR)) represents a potential alternative to the loss of resistance (LOR) technique.

## 2. Methods

### 2.1. 3D Printing

A 3D printable model was created with Fusion360 (Autodesk Inc., San Rafael, CA, USA) software and saved in a stereolithography file format (STL). The STL file was converted into a G-code file containing a series of thin layers with ideaMaker 3.3.0 for fused deposition modeling (FDM) printing and A-series desktopslicer 0.9.1 for stereolithography (SLA) printing. In FDM printing, Raise 3DPro2 plus (Raise3D, Irvine, CA, USA) was used to create an epidural space finder model using a 1.75 mm polylactic acid (PLA) filament (Raise3D, Irvine, CA, USA). The print settings were 0.05 mm of layer height with 0.4 mm nozzle size at a nozzle temperature of 215°C. In SLA printing, Sindoh A1 + SLA (Sindoh, Seoul, South Korea) was used to print the 3D model using ABS-like resin (Sindoh, Seoul, South Korea). The print settings were 0.1 mm layer height and 405 nM laser wavelength.

### 2.2. Biocompatibility Test (Sterility and Extractable Substance)

Extractable substance and sterility tests were performed at KBIO Health and Medical Device Development Center (KBIO Osong Medical Innovation Foundation, Chungbuk, South Korea). The two gears of the 3D print models in PLA or Resin were sterilized with Ethylene oxide (EO) gas and sent to the test facility.

The elution test was performed and analyzed according to the dissolution test and the heavy metal tests developed by the Korean Pharmaceutical Plastic Container Test Method [11].

For the preparation of the test substance, distilled water was used as extractant, and 4.0 g of the sample was eluted at 37 ± 1°C for 72 hours to obtain a sample solution.

#### 2.2.1. Extractable Substances Test

1. Appearance: After elution, 5 mL of the sample solution was placed in a test tube and observed on a white background.
2. pH (acidity and alkalinity): The test used 20 mL of each sample and blank test solution, and 1.0 g of calcium chloride was dissolved in water, to which 1.0 mL of a 1000 mL solution was added. The pH of the two solutions was measured.
3. Reducing substance: A potassium permanganate reduction test was conducted to determine the presence of organic or inorganic substances in the elution solvent using potassium permanganate, a powerful oxidizing agent. A 10 mL of the sample solution in a stoppered conical flask was mixed with 1 mL of dilute sulfuric acid, followed by the addition of 20.0 mL of 0.002 mol/L potassium permanganate solution. The mixture was boiled for 3 minutes, cooled, mixed with 0.10 g of potassium iodide, stirred well, shaken, and left for 10 minutes. The reaction was titrated with 0.01 mol/L sodium thiosulfate. The same procedure was repeated using 10.0 mL of blank test solution. The difference in the consumption of 0.002 mol/L potassium permanganate solution of the sample and the blank test solutions was calculated.
4. Absorbance: Ultraviolet absorption tests were conducted to identify unknown substances with invisible ultraviolet light. The absorbance of the test solution was analyzed using the blank test solution as a control, and the maximum absorbance at each interval of 250–350 nm was recorded.
5. Evaporation residue test: an evaporation residue test was conducted to determine whether an unknown substance was eluted in the eluting solvent. A 10 mL of the sample solution was taken and evaporated, and the residue was dried at 105°C for 1 hour and the difference in weight before and after evaporation was calculated.
6. Heavy metal tests: Heavy metal tests were conducted to determine whether heavy metals (unknown divalent heavy metal ions) were eluted. After taking 10 mL each of the sample and blank test solutions in a Nessler tube, 2 mL of lead standard solution (10 ppm) was added to the blank. Three drops of sodium sulfide and 2 mL of diluted acetic acid were added to the sample and blank solution, and the solution colors were compared on a white background.

#### 2.2.2. Sterility Test

Sterility tests were conducted according to ISO 11737-2. An anaerobic strain (*Clostridium sporogenes*), two fungi (*Candida albicans*, *Aspergillus brasiliensis*), and three aerobic strains (*Staphylococcus aureus*, *Bacillus subtilis*, and *Pseudomonas aeruginosa*) were used. Anaerobic strains were cultured in fluid thioglycollate medium (FTM), whereas fungal and aerobic strains were grown in tryptic soy broth (TSB). A medium performance test was conducted before the main test. FTM was inoculated with *C*. *sporogenes*, *P*. *aeruginosa*, and *S*. *aureus* at 32.5 ± 2.5°C. TSB was inoculated with *A*. *brasiliensis*, *B*. *subtilis,* and *C*. *albicans* at 22.5 ± 2.5°C. This test was conducted by direct method. Samples were inoculated up to 10% of the media volume. After inoculation, FTM was cultured at 32.5 ± 2.5°C and TSB at 22.5 ± 2.5°C for at least 14 days.

### 2.3. Anesthesia and Monitoring

Experiments were performed at KBIO Health Animal Testing Center of the Medical Device Development Center (Osong Medical Innovation Foundation, Osong, Chungbuk, South Korea) following approval of the Institutional Animal Care and Use Committee (KBIO-IACUC-2019-115). Two pigs (Cronex, Hwaseong-si, Gyeonggi-do, South Korea) were used for the test (Yorkshire, Male, 38-39 kg).

Healthy animals were observed and quarantined for 7 days. The experiments were conducted using healthy animals after 7 days of acclimation. The animals were fasted a day before the test. Atropine sulphate (Cheil Pharmaceutical, Daegu, Korea) 0.06 mg/kg was intramuscularly injected on the day of the experiment to prevent bradycardia, a side effect of anesthesia.

Zoletil (Birbackorea, Seoul, South Korea) 6 mg/kg and Rompun (Bayer Korea, Ansan, Korea) 2 mg/kg were injected intramuscularly for anesthesia induction. After airway intubation, general anesthesia was maintained with 3% isoflurane (Fabius GS premium, Drager, Germany).

During anesthesia, electrocardiography (ECG), oxygen saturation, and expiratory carbon dioxide concentration were measured with a Vista 120 monitoring system (Drager, Germany). The animal was laid in the prone position and the limbs were fixed. Radiography was then performed using an angiography machine (Artis Zee Multi-Purpose, Siemens, Germany).

After animal experiments, animals were euthanized by inducing cardiac arrest through intravenous infusion of large amounts of KCl through a catheter mounted in the vein of the ear under anesthesia (5% or more of isoflurane).

### 2.4. Porcine Experiment

The porcine experiment for epidural space analysis was conducted in two piglets by a single anesthesiologist. A PLA model, printed with an FDM 3D printer, was sterilized with EO gas and used in the experiment. The interlaminar space between L1-2 and L4-5 was identified radiographically and marked at each level. The guidewire and 14-gauge Tuohy needle, adapted from the spinal cord stimulator trial kit (Medtronic inc, Minneapolis, MN, USA), were used. The two methods including LOR and LOGR (epidural space finder printed with 3D printer) were used randomly and sequentially to access the epidural space via a paramedian approach at each level. A Touhy needle was located and targeted under fluoroscopic guidance. Immediately after penetrating the thick skin of the pig, the needle trajectory from the skin to epidural space was estimated under fluoroscopic guidance. Immediately after skin penetration, the needle was advanced 1 cm.

When LOR technique was used to identify the epidural space, as the needle was advanced through ligamentum flavum, a constant pressure was applied on the syringe until loss of resistance occurred. The plunger of the syringe was gently withdrawn to assess the dural puncture. To confirm the epidural space entry, the guidewire was advanced a few centimeters into the epidural space.

When LOGR (epidural space finder) was used, the guidewire replaced the air in the LOR technique. While advancing the needle through ligamentum flavum, adequate force was exerted to ensure constant rotation of dials on both sides of the epidural space finder to generate resistance on the guidewire of the device. When the needle entered the epidural space, the dial lost the resistance and the guidewire moved a few centimeters ahead smoothly into the epidural space. After the procedure was completed, the epidural space finder and guidewire were removed, and a glass syringe was connected to a Touhy needle. The plunger of the syringe was gently withdrawn to determine the dural puncture.

The epidural space approach was confirmed by advancing the guidewire and radiographic image. The time taken to advance the needle by 1 cm from the skin to the epidural space was recorded. The depth from the skin to epidural space was measured using the radiographic image. If more than three attempts were made to identify the epidural space, it was marked as a failed attempt and the procedure was stopped to minimize the animal damage.

### 2.5. Statistical Analysis

Data were analyzed using the Excel program (Microsoft Corporation, Seattle, WA, USA) and RexSoft (2018, Rex: Excel-based statistical analysis software. URL http://rexsoft.org/). The tests for normal distribution were performed using Shapiro-Wilk test for small sample sizes. The means of data were compared with the paired sample *t*-test. When the *p*-value is less than 0.05, it is considered statistically significant.

## 3. Results

**Figure 1:**
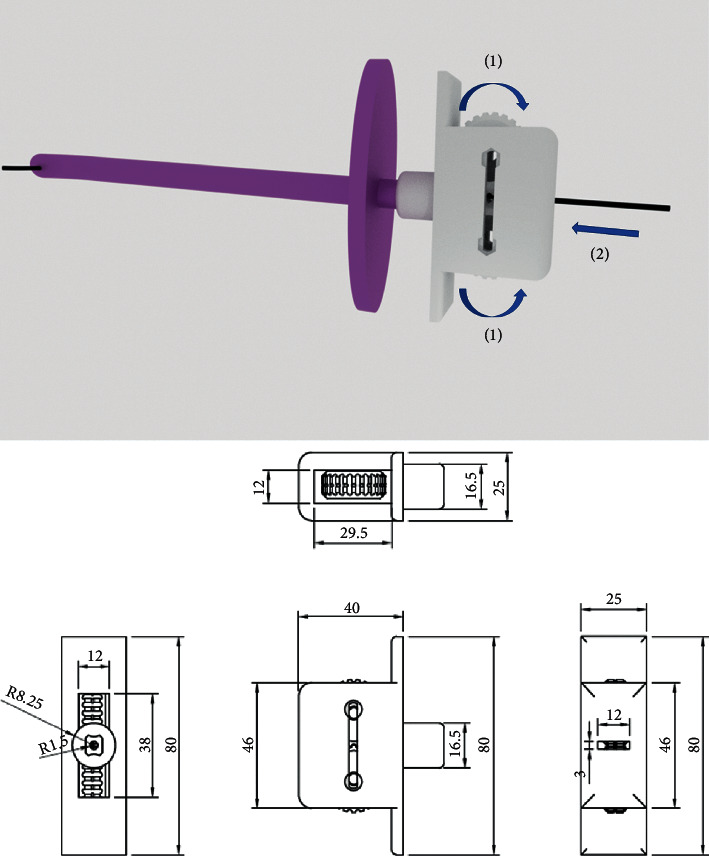
The basic concept of operation in the epidural space finder (up) and blueprint for epidural space finder (bottom) (dimensions in mm).

The basic concept of operation involving the new epidural space finder can be explained as follows. While the needle advances into the lumbar epidural space, a turning force is exerted on the dials ((1) in Figure 1) on both sides of the instruments to continue pushing the wire into the Tuohy needle ((2) in Figure 1). When the needle entered the epidural space, the resistance of the dials is released, and the guidewire moved forward smoothly.

**Figure 2:**
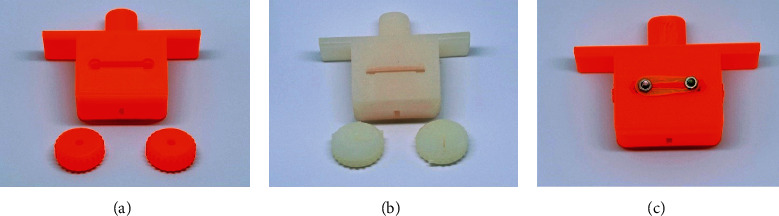
3D printed epidural space finder (PLA product (left), resin product (middle), and assembled epidural space finder (right)).

Different types of 3D printing materials are available, such as acrylonitrile butadiene styrene (ABS), photopolymer resin, polylactic acid (PLA), polypropylene, high-density polyethylene (HDPE), nylon, and polycarbonate (PC) [1]. The PLA and photopolymer resins were selected for the production test due to their popularity in 3D printing. The epidural space finder consists of two dials and one cage. Each printed part was assembled from two sterilized 35 mm M3 bolts and nuts. The two dials were tightened with a sterilized rubber band (Figures 1 and 2). The epidural space finder made of two materials was used for biocompatibility testing (sterility and extractable substance).

A color change of the eluting solvent was observed in the extractable substance test, which confirmed the plasticizer, lubricant, stabilizer, and pigment from the eluate in both PLA and resin products. The difference in pH between the blank and the test solution was 0.07 in PLA and 0.58 in resin, which is smaller than the test standard indicated in Korean Pharmacopoeia (pH = 1.5) (Table 1).

The difference in the consumption of potassium permanganate solution confirmed the presence of organic or inorganic substances. The difference between the potassium permanganate consumption of the blank and the sample solutions was 0.128 mL in the PLA product but 5.75 mL in resin (the test standard < 2 mL) (Table 1).

In the ultraviolet absorption test used to identify the unknown substance with the ultraviolet light, the differences in absorbance between blank and sample solutions were 0.0002 AU for the PLA product and 3.927 in the resin product (test standard < 0.1 AU) (Table 1).

The results of quantitative evaporation residue test showed that 0.2 mg of PLA product and 12.9 mg of resin product were eluted (the test standard of <1.0 mg) (Table 1).

In order to determine if the heavy metals were eluted from the eluate, the color changes in the heavy metal reaction between the standard and the test solutions revealed no differences in both PLA and resin product (Table 1).

The medium performance test was performed to test the culture media. All the strains were appropriate for FTM and TSB. The color of the medium was changed, which confirmed that all the strains were cultured when inoculated into the selection medium. In this test, after 14 days of incubation, both FTM and TSB medium did not turn white and were deemed negative in PLA and resin product (Table 2).

Based on the results of both experiments, the PLA product was more biocompatible than the resin product.

**Figure 3:**
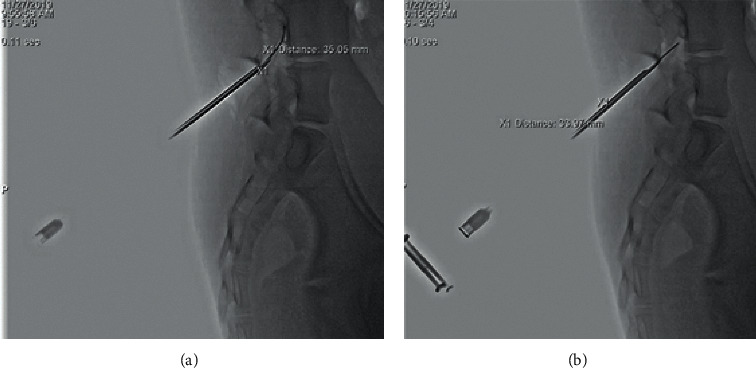
Lateral fluoroscopic image of epidural catheter insertion in porcine experiments: LOR technique (left) and LOGR technique (right).

We decided to use the porcine model to determine the usefulness of the new LOGR compared with LOR technique to identify epidural space. In order to reduce false-positive insertion into epidural space and to minimize animal sacrifice, the needle trajectory into epidural space was estimated under fluoroscopic guidance immediately after penetrating the skin with Touhy needle (Figure 3).

**Figure 4:**
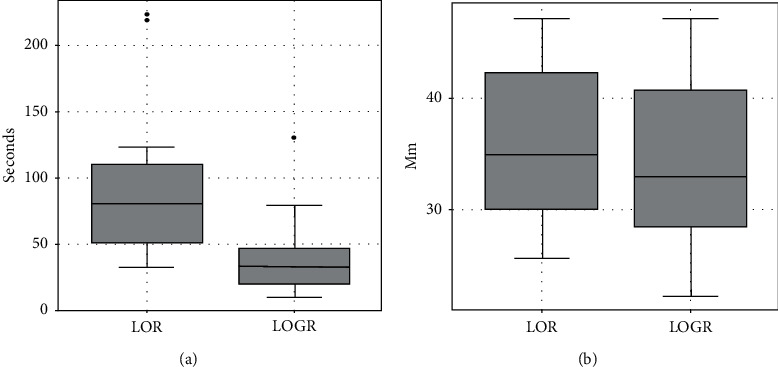
Boxplots comparing the duration of epidural approach (left) and the depth of epidural space (right) between LOR and LOGR techniques.

In two pigs, 14 of 16 epidural insertion attempts were successful via both methods (87.5% success rate). There was no significant difference between the number of attempts in LOGR and LOR groups (1.38 ± 0.81 vs. 1.25 ± 0.68, *p*=0.317, *N* for each group = 14, paired sample sign test). A significant difference was observed in the average time taken for epidural approach between LOGR and LOR groups (41.64 ± 32.18 vs. 92.28 ± 61.46 seconds, *N* for each group = 14, *p*=0.0102 paired sample *t*-test). The mean depth measured from skin to epidural space was not significantly different between LORG and LOR groups (34.14 ± 7.9 vs. 36.25 ± 7.29 mm, N for each group = 14, *p*=0.1174, paired sample *t*-test) (Figures 3 and 4). When the dural puncture was tested by withdrawing the syringe plunger at every attempt, no cerebrospinal fluid (CSF) reflux was observed.

## 4. Discussion

Different techniques are used to identify the location of epidural space during epidural anesthesia or catheter insertion such as LOR, optical spectroscopy, ultrasound, and optical coherence tomography [12]. LOR to air or saline technique is the most frequently used method in common medical practice. Even though LOR technique is simple and easy to perform with a syringe without other complex devices, it requires substantial training to perform correctly and it is not reliable because of its high false-positive rates of up to 30% [13]. In bedside epidural procedures, we frequently use epidural catheter insertion as an adjunct to LOR technique to confirm the true epidural space. When loss of resistance is detected, and if epidural catheter cannot advance through the epidural needle, the tip of the needle may not be in the epidural space. For a long time, we sought to develop a device that combined the advantages of LOR and catheter insertion. After designing several prototypes with 3D printing, we successfully created a new LOGR device (epidural space finder) to identify epidural space.

Our newly developed device is in direct contact with the epidural guidewire but not with the subject body. After consulting with a biocompatibility specialist at KBIO Health, we decided to perform extractable substance and sterility test but not the cytotoxic test. In the biocompatibility test, the resin released a higher number of unknown organic or inorganic substances than the PLA. Both materials were not certified biocompatible but were generally found in a regular 3D print store.

PLA is a well-known biodegradable and biocompatible polymer, which can be produced from the fermentation of corn or sugarcane. PLA is one of the most successful biopolymers with a diverse range of biomedical applications, such as drug delivery, implants, and tissue engineering [14].

Light-activated resin (photopolymer) contains a monomer and an oligomer cross-linked by light and photoinitiator, which generate free radicals that interact with the monomer to induce the polymerization chain reaction [15]. Toxicity varies with the nature of the resin substance. For example, bisphenol A-glycidyl-methacrylate (BisGMA) is more cytotoxic and genotoxic than triethylene glycol dimethacrylate (TEGDMA) monomers [16].

It is not clear whether the substances released from the resin products are harmful to humans, because no cytotoxic tests such as MTT (3-[4,5-dimethylthiazole-2-yl]-2,5-diphenyltetrazolium bromide) assays were conducted. Even though biocompatible resins are commercially available, it is certain that PLA is safer than the resin, at least in our experiment.

In the porcine model, the LOGR technique was faster than the LOR technique in facilitating access to the epidural space. However, no difference in success rate was observed. We could not completely evaluate the procedure related complications such as dural puncture, because no radiopaque contrast dye was injected after the epidural approach to minimize the porcine sacrifice. We evaluated the CSF reflux after each experiment; however, it is not a reliable method to confirm dural puncture. It is reasonable to conclude that LOGR and LOR techniques are comparable in their ability to provide access to the epidural space. However, it was not clear which technique was associated with fewer complications such as dural puncture or epidural hematoma. Further studies are needed to establish the adverse effects associated with LOGR technique.

Most of the 3D printing applications in anesthesiology to date focus on rapid production, reduced manufacturing cost, and ability to provide a delicate printing design for anesthesia planning or medical training. In addition, we sought to determine the potential of 3D printers in the manufacture of products anywhere in the world outside the factories. Therefore, we tested PLA and resin, the materials used most often for 3D printing. Using these materials in a hospital, we can print our own devices with an inexpensive 3D printer.

In summary, a 3D printer can be used to develop new medical devices for anesthesia and can also be used as an effective substitute when the normal supply chain collapses such as in a war, earthquake, or typhoon.

## Figures and Tables

**Table 1:** Results of extractable substance test (^*∗*^: test standard value of Korean Pharmacopoeia [11]).

	PLA-FDM	Resin-SLA
Appearance (clear^*∗*^)	Clear	Clear
pH (<1.5)	0.07	0.58
Reducing substance (<2 mL)	0.1282 mL	5.75 mL
UV absorbance (<0.1)	0.002	3.927
Residue on evaporation (<1.0 mg)	0.2 mg	12.9 mg
Heavy metal (negative)	Negative	Negative

**Table 2:** Results of sterility test.

	PLA-FDM	Resin-SLA
Fluid thioglycollate medium (FTM)	No growth	No growth
Trypticase soy broth (TSB)	No growth	No growth

## Data Availability

The data used in the study are available on request.

## Abbreviations

PLA: Polyactic acid
LOR: Loss of resistance
LOGR: Loss of guide wire resistance
FDM: Fused deposition modeling
SLA: Stereolithography
FTM: Fluid thioglycollate medium
TSB: Tryptic soy broth
ABS: Acrylonitrile butadiene styrene (ABS)
PLA: Polylactic acid
HDPE: High-density polyethylene
PC: Polycarbonate
CSF: Cerebrospinal fluid
BisGMA: Bisphenol A-glycidyl-methacrylate
TEGDMA: Triethylene glycol dimethacrylate.
